# Plant-Based Diets Reduce Blood Pressure: A Systematic Review of Recent Evidence

**DOI:** 10.1007/s11906-023-01243-7

**Published:** 2023-05-13

**Authors:** João Tomé-Carneiro, Francesco Visioli

**Affiliations:** 1 Food Bioactive Ingredients, IMDEA-Food, Madrid, 28049 Spain; 2 Department of Molecular Medicine, University of Padova, Viale G. Colombo 3, 35121 Padova, Italy

**Keywords:** Plant-based diets, Hypertension, Polyphenols, Cardiovascular disease, Vitamin C, Essential fatty acids

## Abstract

**Purpose of Review:**

Accumulating data on the consumption of plant-based diets and their impact on blood pressure indicate a consensus that plant-based diets are linked to reduced blood pressure. The suggested mechanisms of action are manifold, and, in this systematic review, we provide a summary of the most recent findings on plant-based diets and their impact on blood pressure, along with an analysis of the molecules accountable for the observed effects.

**Recent Findings:**

The overwhelming majority of intervention studies demonstrate that plant-based diets result in lower blood pressure readings when compared to diets that are based on animal products. The various mechanisms of action are being clarified.

**Summary:**

The data discussed in this systematic review allow us to conclude that plant-based diets are associated with lower blood pressure and overall better health outcomes (namely, on the cardiovascular system) when compared to animal-based diets. The mechanisms of action are being actively investigated and involve many macro- and micronutrients plentiful in plants and the dishes prepared with them.

**Supplementary Information:**

The online version contains supplementary material available at 10.1007/s11906-023-01243-7.

## Introduction

Data concerning the intake of plant-based diets and their effects on blood pressure keep accumulating, and there is consensus that plant-based diets are associated with lower blood pressure. The proposed mechanisms of action are manifold, and, in this systematic review, we summarize the latest data on plant-based diets and blood pressure, with insights on the molecules responsible for the described effects.

We first want to briefly highlight two of the major cross-sectional studies available on this topic. On the one hand, the Epic-Oxford study included more than ten thousand British subjects and showed that vegans presented the lowest levels of hypertension and blood pressure (BP), while meat-eating individuals presented the highest ones [1••]. Likewise, in arbitrarily selected subjects from the Adventist Health Study-2 (AHS-2) cohort (comprising almost one hundred thousand Adventists in North America), vegans and vegetarians presented lower BP levels than the individuals eating meat [2]. Noteworthy, in both studies, adjustment for body mass index (BMI) reduced the magnitude of these findings.

The impact of dietary patterns on BP has also been explored in numerous prospective cohort investigations. In the context of the Coronary Artery Risk Development in Young Adults (CARDIA), a dose-dependent relationship was found between plant food intake (whole grains, fruits, vegetables, etc.) and reduced incidence of raised BP [3]. In addition, in the combined analysis of the Nurses’ Health Study (NHS) I and II and the Health Professionals Follow-up Study (HPFS), an association was found between hypertension and the consumption of meat and seafood [4]. Similar findings were reported in other smaller prospective studies [5-8].

## Methods

**Fig. 1:**
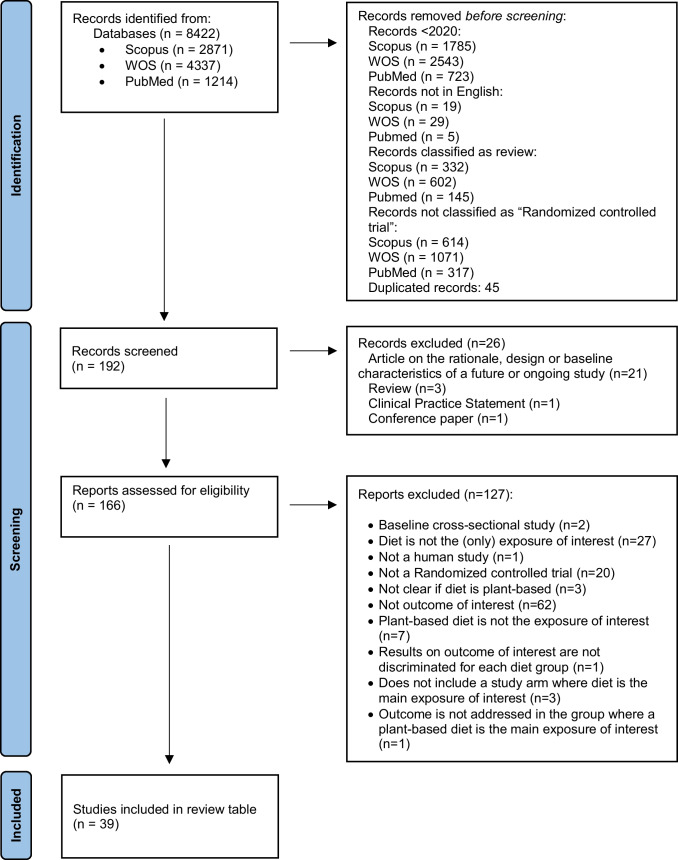
PRISMA 2020 flow diagram of plant-based diets studies (as related to blood pressure) retrieved

The search strategy — according to the Preferred Reporting Items for Systematic reviews and Meta-Analyses (PRISMA) [9] — is shown in Fig. 1. We searched PubMed, Web of Science, and Scopus. Literature search strategies used for each database and the syntax search terms are displayed in Supplementary Table 1. The search retrieved 8422 articles. After removing papers < 2020, duplicated records, reviews, non-human studies, etc., we review 39 papers, discussed in Table 1.

**Table 1:** Recent studies on plant-based diets and blood pressure

**Volunteers** (*n*, age)	**Healthy/pathological context**	**Study arms/groups**	**Study design** **Intervention period**	**Main results on BP/hypertension**	**Ref**
62, 56.6 ± 10.9 y, group 1; 58.3 ± 8.4 y, group 2	Overweight	(1) Med diet; (2) vegan diet. The Mediterranean diet followed the PREDIMED protocol. The low-fat vegan diet (∼75% of energy from carbohydrates, 15% protein, and 10% fat) consisted of vegetables, grains, legumes, and fruits. Participants were instructed to avoid animal products and added fats. Vitamin B_12_ was supplemented (500 µg/day) during the vegan phase of the study	Crossover. 16 weeks on diet 1 + 4 weeks on baseline diet + 16 weeks on diet 2	SBP and DBP decreased when on the Med diet (− 9.3 and − 7.3 mmHg) and vegan diet (− 3.4 and 4.1 mmHg) (treatment effect + 5.9 [95% CI + 1.0 to + 10.9]; *P* = 0.02; and + 1.8 [95% CI − 4.6 to + 8.1]; *P* = 0.58, respectively)	[53]
5373, 55–75 y	Overweight/obese with MetS	Control group: Med diet supplemented with EVOO and nuts, without caloric restriction or promotion of PA or weight loss goals. Intervention group: hypocaloric Med diet (with a caloric restriction of 30%) supplemented with EVOO and nuts, as well as an intensive lifestyle program with promotion of physical activity and weight loss goals including behavioral therapy	Parallel. 12 months	After 6 and 12 months, SBP and DBP decreased across successive quintiles of improvement in the carbohydrate quality index (categorized in quintiles and based on 4 criteria: total dietary fiber intake, glycemic index, whole grain/total grain ratio, and solid carbohydrate/total carbohydrate ratio)	[28]
187, 49 ± 10 y	Overweight, moderate-to-severe obstructive sleep apnea	(1) Standard care group (SCG, *n* = 65), (2) Med diet group (MDG, *n* = 62), or (3) Med lifestyle group (MLG, *n* = 60). Intervention arms were subjected to a 6-month weight-loss behavioral intervention based on the Med diet	Parallel. 12 months	BP improved only in intervention arms and was significantly lower vs. SCG post-intervention. MLG had a lower relative risk of hypertension (MDG only showed a trend) compared to SCG	[31]
2278, 59.6 ± 2.1 y (Q1); 74.2 ± 2.6 (Q4)	High CVD risk, but no CVD at enrolment	A traditional Med diet supplemented with either (1) complementary EVOO or (2) tree nuts, or (3) a control diet based on advice to follow a low-fat diet	Parallel. 3-year follow-up	SBP levels decreased in both quartiles (change was greater in the Q1). DBP decreased in Q4, contrary to Q1 (BP increased significantly)	[166]
50, median (IQR): 62.8 (53.9, 70.8)	Rheumatoid arthritis	(1) Intervention diet group or (2) a control diet group. Intervention diet was rich in whole grain, fatty fish, nuts, vegetables, and fruit and supplemented with probiotics. The control diet resembled average nutritional intake in Sweden	Crossover. 10 weeks + a 4-month wash-out	SBP and DBP values showed a tendency for decreasing from baseline in both groups, but the difference between groups was not significant	[46]
70, 61.1 ± 7.0y, PBDG; 62.8 ± 7.0 y, CG	Cardiometabolic risk	(1) Ad libitum whole-food plant-based diet group, consisting of vegetables, grains, legumes, and fruits and avoidance of animal products (PBDG, *n* = 36) or (2) control group (CG) which continued an omnivorous diet (*n* = 34)	Parallel. 8 weeks	Ambulatory BP monitoring was not significant between the groups, although PBDG showed more favorable effects for SBP and DBP vs controls	[55]
60, median (range): 63.5 (51–75), A; 60.0 (44–74), B; 61.0 (34–74), C	T2DM	Investigational diet group (A) was compared with a food exchange system‐based diet, either in the form of ready meals provided to participants (group B) or not (group C). The investigational diet mimicked the average East Asian diet, but retained common characteristics of the Med and DASH diets	Parallel. 12 weeks	Reductions in SBP (− 1.90 ± 11.41 in group A vs 7.95 ± 11.92 in group C, *P* = 0.011) and DBP (− 1.60 ± 7.96 in group A vs 5.00 ± 9.85 in group C, *P* = 0.025) between baseline and week 12 were greater in A vs. C	[52]
10371, 50–79 y	Normotensive postmenopausal women without prior history of CVD	(1) Intervention group (*n* = 4245), (2) comparison group (*n* = 6126). The dietary goals of the intervention group included a reduction in total fat intake to 20% of total energy, an increase in vegetable and fruit intake to 5 servings/d, and an increase in grain intake to 6 servings/d	Parallel. 8.3 years	Small reductions in BP were observed in the intervention group	[62]
277, 48 ± 9 y	Moderate abdominal obesity or/and dyslipidemia	(1) Med/low carbohydrate diet (MED/LC, *n* = 80) or (2) low fat (LF, *n* = 79) iso-caloric weight loss intervention diet. The MED/LC diet consisted in high protein and fat intake, mostly from vegetarian sources, according to the Med diet; the LF diet was based on the AHA guidelines	Parallel. 18 months	At 6 months, a statistically significant reduction was found in both DBP and SBP in participants in the low RQ group consuming the MED/LC diet	[35]
208, 48.8 ± 11.8 y	T2DM	Intervention group (IG, *n* = 104) or control group (*n* = 104). Both had usual medical care. IG also received extended nutritional advice, including instructions to eat meals with one-half of the plate consisting of non-starchy vegetables, one-quarter with lean protein, and one-quarter with low glycemic index carbohydrates such as whole grains	Parallel. 6 months	Significant decreases in BP from baseline were seen in both groups, but changes were significantly higher in the intervention group	[61]
250 families (n = 720), 3-85y	Without major CVD	(1) Intervention group (127 families, *n* = 367) received educational sessions, cooking classes, written supporting material, and foods that form part of the Atlantic diet. (2) Control group (123 families, *n* = 353) followed their habitual lifestyle. The Atlantic diet has several characteristics in common with the Med diet	Parallel. 6 months	Non-significant intra- and inter-group differences in SBP and DBP	[49]
40, 48.6 ± 7.6, controls; 51.9 ± 6.2, J-DASH 1; 50.0 ± 9.5, J-DASH 2	Untreated high normal BP or stage 1 hypertension	J-DASH 1 and J-DASH 2 groups (*n* = 13 each) and the usual-diet group (*n* = 14). The J-DASH diet was in accordance with the nutritional composition of the DASH diet, but contained less total fat and saturated fatty acids	Parallel. 6 months: 2-month intervention + 4-month follow-up	J-DASH diets improved home measured BP and stabilized its variability compared to the usual diet	[51]
235, 40–65 y	Moderate obesity	Secondary study of the DIRECT trial. (1) Low-fat, restricted-calorie diet (*n* = 85), (2) Med, restricted-calorie, diet (*n* = 76), or (3) low-carbohydrate, non–restricted-calorie diet (*n* = 74). Med diet and low-fat diet volunteers were counseled to consume low-fat grains, vegetables, fruits, and legumes and limit consumption of additional fats, sweets, and high-fat snacks. The low-carbohydrate, non–restricted-calorie diet was adapted from the Atkins diet	Parallel. 24 months	Significant reductions in SBP for all diet groups at both 6 months and 24 months (all P < 0.05 for within-group comparisons), and significant reductions in DBP for the Med diet group (P < 0.05 at both time points)	[36]
44, mean (95%CI): 50.73 (48.65, 52.8)	MetS	(1) Med diet or (2) non-Med diet + nuts (50 g/day)	Crossover. 2 months + a 1-month wash-out	Non-significant intra- and inter-group differences in SBP and DBP	[30]
71, 58 ± 8y (Fast + DASH) and 62 ± 8y (DASH)	CVD risk patients with MetS	(1) After a 5-day fasting patients were instructed to follow a modified DASH diet (Fast + DASH diet (*n* = 35)), with additional emphasis on plant-based and Med diet to optimize refeeding, or (2) DASH diet (*n* = 36)	Parallel. 12 weeks	DASH reduced office SBP; Fast + DASH led to a sustained reduction in 24 h ambulatory SBP and MABP. Fast + DASH and DASH reduced intake of antihypertensive medication in 43% and 17% of cases, respectively	[167]
253, 38 ± 8, 1; 37 ± 9, 2; 38 ± 9, 3	Prediabetes	3 isocaloric-restricted diets: (1) Med diet, (2) a traditional Jiangnan diet high in plants, or (3) a control diet low in plants	Parallel. 6 months	SBP and DBP decreased significantly from baseline for all diets but differences between groups were non-significant	[40]
43, 65 ± 15y	Chronic kidney disease	(1) Med diet, (2) vegetarian diet (very-low-protein diet or VLPD), (3) Med diet supplemented with one tablet of essential amino acids and ketoanalogs	Crossover. 6-month intervention + a 3-month washout	VLPD lowered both the SBP and DBP more effectively than Med diet or KA-supplemented Med diet (*P* < 0.05 in both cases)	[54]
6636, 55–75 y (men) and 60–75 (women)	MetS	Intensive intervention group or control group. Intervention consisted of an energy-reduced Med diet with enhanced physical activity, behavioral support and weight loss compared to an energy-unrestricted Med diet (control)	Parallel. 1 year	A 1-SD increase in the DASH diet score was significantly associated with lower SBP (− 0.57 mmHg, *P* < 0.001) and DBP (− 0.15 mmHg, *P* = 0.03)	[50]
108, 75 ± 6y	Apparently healthy	(1) Experimental group maintained a dietary pattern based on the traditional Cretan Med diet (i.e., vegetables, fruits, olive oil, legumes, fish, whole grain cereals, nuts and seeds and low consumption of processed foods, dairy products, red meat, and vegetable oils). (2) Control group maintained their customary lifestyle and diet	Parallel. 6 months	The Med diet resulted in improved SBP in comparison with the habitual diet. At 18 months (12 months after the end of the intervention study), this improvement was not sustained	[33]
164, 59 ± 8.6, vegan group; 58 ± 11.7 y, vegetarian group	T2DM	(1) Vegan (*n* = 81) or (2) vegetarian (*n* = 83) diets. A low-carbohydrate vegan diet, high in canola oil and plant proteins, or a vegetarian therapeutic diet	Parallel. 3 months	No changes in BP medications and no treatment differences in BP. Both diets had significant reductions from baseline in SBP and DBP: − 4.12 and − 3.54 mmHg, respectively (vegan) and − 5.91 and − 4.13 mmHg, respectively (vegetarian)	[56]
6932, 66–68y on average	Overweight/ obese with MetS	Four sub-groups of PREDIMED participants: (1) nonusers (*n* = 2188), and users of (2) one (*n* = 2401), (3) two (*n* = 1702), or (4) three (*n* = 641) hypertensive drugs	Parallel. 5 years	Nontreated participants on a Med diet had decreased need for antihypertensive drugs. Med diet decreased the need of escalating therapy in patients using 2 drugs at baseline and attenuated the association of drug use with the risk of incident cardiovascular events	[25]
5800, 55-75y	Overweight/ obese with MetS	Control group: Med diet supplemented with EVOO and nuts, without caloric restriction or promotion of PA or weight loss goals. Intervention group: hypocaloric Med diet (with a caloric restriction of 30%) supplemented with EVOO and nuts, as well as an intensive lifestyle program with promotion of PA and weight loss goals including behavioral therapy	Parallel. 1 year	As nut consumption increased a significant decrease was found in SBP (*P* < 0.05)	[29]
41, median (range): 58 (33–65), Med Diet; 58.5 (27–65), low-fat diet; 53 (25–67), Controls	Clinically stable heart or lung transplant recipients at least 6 months post-surgery	(1) Med diet, (2) low-fat diet. A Med diet supplemented with EVOO, or a low-fat diet based on modified British Heart Foundation dietary guidelines. The control group consisted of eligible transplant recipients who did not take part in the trial and remained clinically stable at points corresponding to trial baseline and end	Parallel. 12 months	SBP and DBP increased in the control group, whereas, compared with the Med group, the low-fat group showed a greater decline in these parameters. In the Med group, 4 patients moderately decreased their antihypertensive medication dose	[11]
138 women, 47.2 ± 11.3 y	Obese	Participants were assigned to the almond-supplemented Med diet group (MDSA) or maintenance of usual diet (control) group in which no changes in dietary habits were advised	Parallel. 3 months	SBP and DBP decreased in MDSA and control groups, with no significant differences between groups	[37]
60, 42.4 ± 6.8 y	Stable HIV infection	Diet1 group received dietary advice to reduce saturated fat intake to < 10% of energy intake. Diet2 group were supported to adopt the Med Portfolio Diet (Diet2) with additional cholesterol-lowering foods (nuts, stanols, soya, oats, beans)	Parallel. 6 months	Diet2 participants had a significantly lower SBP (mean difference adjusted for baseline − 7 mmHg, 95% CI − 2 to − 12, *P* = 0.008) compared to those in Diet1	[45]
21, 22 ± 4 y	Professional female handball players	(1) Free diet; (2) Med diet; or (3) High antioxidant diet	Parallel. 12 weeks	No significant differences were found in BP over time or between groups	[48]
15, 36.1 ± 10.0 y	T1DM	Three isocaloric dietary patterns: (1) high-protein/low-carbohydrate diet with 20% of calories as carbohydrates, (2) Med/low glycemic index diet with 40% carbohydrates, (3) a reference diet with 50% carbohydrates	Crossover. 3 weeks, with 7-day washout periods	No significant differences were observed in BP changes between groups	[41]
66, children (3–9 y), adolescents (10–19 y), adult (≥ 20y)	Living in a situation of extreme poverty	(1) Control group, (2) intervention group. More healthy food items such as fruits and vegetables, legumes, fish, nuts and seeds, and olive oil were consumed in the intervention group compared to the control group	Parallel. 10 weeks	BP did not improve in either the intervention or the control group	[63]
70 adolescent women, 14 ± 1y	MetS	(1) Intervention group, (2) control group. The intervention group followed a prescribed Med diet, while participants in the control group received dietary advice according to the food pyramid	Parallel. 12 weeks	Significant reduction in SBP (− 8.4 ± 1.4 vs. 1.4 ± 1.4 mmHg, *P* < 0.001) in Med diet vs. control diet. No significant effect on DBP	[39]
369, 64.57 ± 8.95, BALANCE; 64.11 ± 9.65, Control	CVD events in the previous 10 years	(1) BALANCE Program group, (2) control group. Control group: low-fat, low-energy, low-sodium, low-cholesterol diet; the BALANCE Program group: diet with a nutritional composition based on the Med and DASH diets	Parallel. 6 months	Decreases in SBP and DBP in BALANCE (mean (mmHg), − 6.0 and − 5.34, respectively)) and in control (− 8.4 and − 6.59, respectively) groups, with non-significant differences between groups	[38]
366 women, 46.9 ± 11.1 y	BRCA1/2 mutations, with or w/o diagnosis of BC/OC and w/o metastases	Active dietary intervention group (IG) or control group (CG)	Parallel. 6 months	LOF variant carriers: decrease in SBP in IG and CG was equivalent (Δ = − 0.1 mmHg). In nonsynonymous variant carriers, SBP remained almost unaltered in IG, while an increase in CG (Δ = − 3.1 mmHg; *P* < 0.01) was observed	[47]
96 children, 9 to 18 y	BMI > 95% age/sex predicted	(1) Plant-based diet consisting of only whole foods, including fruits, vegetables, beans, other legumes, and whole grains, limited salt, avocado and nuts, and no-added-fat. (2) AHA diet encourages eating plant-based whole foods, and low-sodium intake but permits some non-whole foods, low-fat dairy, selected plant oils, lean meat, and fish in moderation. (3) Med diet was similar to AHA (but with more emphasis given to fish and EVOO and/or nuts)	Parallel. 52 weeks, follow-up at weeks 4 and 52	All diets were associated with similar statistically significant (*P* < 0.05 to < 0.001) improvements in SBP and DBP. Significant improvements in all groups at week 4 in SBP and DBP. Significant improvements at 52 weeks in both the AHA and the plant-based diet groups in SBP and DBP	[60]
53, 52.8 ± 6.8y (fast + PBD) and 51.2 ± 11.5 (DGE diet)	Rheumatoid arthritis	(1) 7-day fast followed by an 11-week plant-based diet (fast + PBD) or (2) 12-week standard Deutsche Gesellschaft für Ernährung (DGE) diet	Parallel. 12 weeks	Neither dietary protocol lowered SBP, although there was a tendency to decrease DBP in the fast + plant-based diet group	[59]
149, 39.63 ± 8.82	Severely obese individuals (BMI ≥ 35.0 kg/m^2^)	(1) 52 mL/day of EVOO, (2) DieTBra, or 3) DieTBra + 52 mL/day of EVOO. Traditional Brazilian Diet (DieTBra): plenty of tropical fruits; raw and cooked vegetables and legumes (lunch and dinner); bread, coffee, and milk (breakfast); moderate consumption of dairy products; small portion of red meat; rare consumption of seafood and nuts; rice and beans (lunch and dinner); predominant use of soy oil	Parallel. 12 weeks	In the DieTBra + EVOO group, DBP (0.053) and SBP (*P* = 0.072) decreased without statistical significance. No differences between groups	[64]
75, mean: 57.1 y	Low Med diet adherence, overweight, and at high CVD risk	(1) A community-based peer support intervention, (2) dietitian-led intervention, or 3) control group. (1) A community-based peer support intervention to encourage adherence to Med diet compared to a (2) dietitian-led intervention and a (3) minimal support intervention (control group)	Parallel. 12 months	No statistically significant differences between intervention groups. SBP and DBP were significantly different over time in the study population as a whole	[34]
710, median (IQR): 57 (46, 63)	Overweight/obese and pre-diabetic	Available-case analysis where all participants were merged into 1 group to assess longitudinal associations of adherence to an overall plant-based diet and plant food intake with yearly changes. (1) high protein diet and high-intensity PA, (2) moderate protein diet and high-intensity PA, (3) high protein diet and moderate-intensity PA, (4) moderate-protein diet and moderate-intensity PA	Parallel. 8-week weight loss plus a 148-week intervention	Fruit and vegetable intake was inversely associated with increments in DBP. No associations between an overall plant-based diet and BP after adjustment for weight change	[168]
63, 48.0 ± 10.6y	Risk of T2DM	Healthy US group (HUS, *n* = 21), Med group (Med, *n* = 22), vegetarian group (Veg, *n* = 20). Dietary patterns according to USDA dietary guidelines	Parallel. 12 weeks	SBP and DBP decreased in all groups (− 5.5 ± 2.7 mmHg HUS, − 3.2 ± 2.5 mmHg Med, − 2.4 ± 2.9 mmHg Veg; statistical significance only in HUS), yet changes were not significantly different between groups	[169]
98, 51.1 (9.8) y (AHA group) and 49.2 (8.9) y (FLiO group)	Overweight/obese subjects with NAFLD	(1) American Heart Association (AHA) dietary group or (2) the FLiO dietary group. The FLiO diet proposed a high adherence to the Med diet	Parallel. 2 years	DBP decreased significantly in both groups and SBP also decreased (statistical significance only in AAH diet). In both cases, values were not significantly different between groups	[44]
228, 57.3 ± 9.28 y	T2DM	(1) Control group or (2) Med diet educational intervention group	Parallel. 6 months	SBP and DBP decreased significantly in comparison with the control group (*P* < 0.001 and *P* = 0.04, respectively)	[42]

*AHA* American heart association, *BALANCE* Brazilian Cardioprotective Diet Program, *BC/OC* breast cancer and/or ovarian cancer, *BMI* body mass index, *BP* blood pressure, *BRCA1/2* breast cancer gene 1/2, *CI* confidence interval, *CVD* cardiovascular disease, *DASH* Dietary Approaches to Stop Hypertension, *DBP* diastolic blood pressure, *DieTBra* traditional Brazilian diet, *DIRECT* diabetes remission clinical trial, *EVOO* extra virgin olive oil, *FLiO* fatty liver in obesity study, *IQR* interquartile range, *J-DASH* Japanese cuisine-based DASH, *LOF* loss-of-function, *MABP* mean arterial blood pressure, *Med* Mediterranean, *MetS* metabolic syndrome, *NAFLD* non-alcoholic fatty liver disease, *PA* physical activity, *SBP* systolic blood pressure, *T1/2DM* type 1/2 diabetes mellitus, *USDA* United States Department of Agriculture, *VLPD* very low protein diet, *y* years

### Evidence on the Effects of Particular Plant-Based Diets

Many types of dietary patterns exist in the arena of essentially plant-based diets, including vegetarian diets and their variants, the Dietary Approach to Stop Hypertension (DASH) diet, the Mediterranean (Med) diet, the healthy Nordic diet, and many other high-fruit, vegetable, and whole grain diets.

#### Vegetarian Diets

Vegetarian diets usually do not include meat, poultry, or fish, although individual eating patterns do vary. Lacto-ovo-vegetarian diets are based on grains, vegetables, fruits, legumes, seeds, nuts, dairy products, and eggs, excluding meat, poultry, and fish. The lacto-vegetarian diet also excludes eggs besides meat, poultry, and fish. The vegan, or pure vegetarian diet not only excludes meat, poultry, or fish, but also dairy and additional animal products. A meta-analysis of 32 cross-sectional studies, including more than twenty-thousand individuals, indicated that a lower mean BP was related with the intake of vegetarian diets compared to omnivorous ones [10]. Accordingly, a recent meta-analysis, comprising a total of 187 participants from five controlled trials, showed a mean reduction in systolic blood pressure (SBP) (−5.47 mmHg (95% CI, −7.60, −3.34); *P* < 0.00001) and diastolic blood pressure (DBP) (−2.49 mmHg (−4.17, −0.80; *P* = 0.004) in subjects using a lacto-ovo vegetarian diet compared with the consumption of comparator diets, with a high certainty of the results for SBP and a moderate one for DBP [11]. Furthermore, a meta-analysis, which included 856 subjects from 15 randomized controlled trials (RCTs), showed that vegetarian diets significantly reduced SBP (−2.66 mmHg (−3.76, −1.55), *P* < 0.001) and DBP (−1.69 mmHg (−2.97, − 0.41), *P* < 0.001) compared to omnivorous diets [12]. In the subgroup analysis, a vegan diet showed higher decrease in SBP (−3.12 mmHg; (−4.54, −1.70), *P* < 0.001) compared to a lacto-ovo-vegetarian diet (−1.75 mmHg, (−5.38, 1.88), *P* = 0.05). The vegan diet showed a similar trend in terms of DBP reduction (−1.92 mmHg (−3.18, −0.66), *P* < 0.001), while those in a lacto-ovo-vegetarian diet did not. Conversely, another meta-analysis, including 677 individuals from nine controlled trials, showed that the consumption of vegan diets was not significantly associated with a mean reduction in SBP (−1.30 mmHg (−3.90, 1.29); *P* = 0.33) and DBP (− 0.81 mmHg (−2.91, 1.28); *P* = 0.45) compared with the consumption of comparator diets, although the reliability of results was low [13]. Moreover, the authors acknowledge that the use of BP-lowering comparator diets probably underrated the BP-lowering capability of vegan diets. Indeed, when the usual diet of volunteers was used as the comparator, there was a statistically significant overall effect estimate, although reliability remained low.

#### DASH Diet

The DASH randomized trial was designed to evaluate the effects on BP of a fundamentally plant-based diet, which included plant foods, low-fat dairy, and limited amounts of lean meat [14]. During this eight-week trial, volunteers consumed one of the following diets: (1) a “standard” American diet (control), (2) the DASH diet, or (3) a fruits and vegetables diet. Compared to the fruits and vegetables diet, the DASH diet had a reduced fat content and consisted in more daily servings of fruits and grains and fewer meat servings. The DASH diet reduced BP when compared with the control diet, especially in African Americans, and in already hypertensive subjects [15, 16]. The potential impact on BP reduction caused by a plant-based diet was verified in the DASH-sodium trial, which showed that restraining sodium led to further effects on BP reduction in combination with the DASH diet [17] and that higher BP levels at baseline were associated with an enhanced reduction of BP [18]. A recent meta-analysis comprising a total of 1400 participants from 11 controlled trials showed that SBP (−5.53 mmHg (95% CI −7.95, − 3.12); *P* < 0.00001) and DBP (–3.78 mmHg (–5.51 to –2.04); *P* < 0.0001) levels were reduced upon consumption of DASH diets compared with the consumption of comparator diets, with a high degree of certainty of the results [13].

#### Mediterranean Diet

The Mediterranean (Med) diet entails a moderate intake of poultry, eggs, seafood, and dairy products and a low consumption of red and processed meats. In this sense, the Med diet can be considered to a large extent to be a plant-based diet, as it comprises a high consumption of plant-based foods such as fruits, vegetables, legumes, grains, nuts, and olive oil [19]. In a meta-analysis of observational studies, including prospective and cross-sectional (four and eight, respectively) studies, representing over 30,000 individuals and 6342 cases of metabolic syndrome (MetS), BP showed an inverse significant association with high adherence to the Med diet (RR = 0.87; 95% CI: 0.77–0.97) [20]. Several RCTs have estimated the effects of the Med diet on BP, and at least three meta-analyses showed consistent results. The first one, which included six trials, comprising more than 7000 people, found that adopting a Med diet pattern for at least 1 year reduced both SBP (− 1.44 mmHg) and DBP (− 0.70 mmHg) levels [21]. Another meta-analysis included three RCTs focused on different healthy dietary patterns and reported an association between the Med diet and a reduction on SBP (− 3.02 mm Hg) and DBP (− 1.99 mm Hg) [22]. Finally, a more recent meta-analysis, comprising a total of 5276 participants from eight controlled trials, showed that SBP (− 0.95 mmHg; 95% CI, − 1.70, − 0.20); *P* < 0.00001) and DBP (− 0.69 mmHg (− 1.44, 0.06); *P* = 0.07) levels were lessened upon consumption of a Med diet compared with the consumption of comparator diets, with a moderate certainty of the results [13].

#### Nordic Diet

Like the DASH and Med diets, the healthy Nordic diet is essentially a plant-based dietary pattern, consisting of whole grains, fruits, vegetables, legumes, rapeseed oil, fatty fish (e.g., salmon), shellfish, seaweed, low-fat meat (e.g., poultry), and dairy, along with restraining salt and sugar-sweetened products [23]. A recent meta-analysis, comprising a total of 420 participants from three controlled trials, showed that the consumption of a healthy Nordic diet led to a reduction in SBP (− 4.47 mmHg; 95% CI, − 7.14, − 1.81); *P* < 0.001) and DBP (− 2.32 mmHg (− 3.83, − 0.82); *P* = 0.002) levels compared with the consumption of comparator diets, with a moderate certainty of the results [13].

#### Other High-Fruit/-Vegetable/-Fiber Diets

Diets not fitting in the previous categories, but that could still be contained in the plant-based diet arena include the ones characterized by increased consumption of fruit and vegetables and high-fiber diets focused on increasing whole-grain and legume consumption. In a meta-analysis including 140 subjects from two RCTs, high-fruit/high-vegetable diets were linked with reduction in SBP (− 0.57 mmHg (− 7.45, 6.32); *P* = 0.87) and DBP (− 0.96 mmHg (− 3.08, 1.15); *P* = 0.37) compared with the consumption of comparator diets [13]. Similarly, consumption of high-fiber diets resulted in a decrease in SBP (− 0.65 mmHg (− 1.83, 0.53); P = 0.28) and DBP (− 1.02 mmHg (− 3.86, 1.82); *P* = 0.48) compared with the consumption of comparator diets (meta-analysis with 316 subjects from two RCTs) [13]. However, in both cases, the certainty of evidence was extremely low.

In line with the results described above, Dinu and colleagues published the first umbrella review focusing on the effects of different popular diets on cardiometabolic risk factors [24•]. Regarding plant-based diets and BP outcomes, the review included meta-analyses of RCTs assessing the DASH (*n* = 6), Mediterranean (*n* = 11), vegetarian (*n* = 9), and Nordic (*n* = 2) diets, among others. Overall, the findings of this review support that balanced dietary patterns, favoring vegetables, fruits, whole grains, and plant-based protein, and limit sugar, sodium, and red and processed meat, are beneficial in adverse cardiometabolic scenarios. Notably, DASH and, especially, Med diets showed the most consistent findings regarding a reduction in BP accompanied by improvements in other parameters, whereas for the Nordic and vegetarian dietary patterns, only weak evidence of a beneficial effect in BP levels was found. Nevertheless, the authors emphasize that around 80% of the meta-analyses included in the study suffered from low methodological quality and weak strength of evidence and included, for many diets, a reduced number of clinical trials. Furthermore, the authors acknowledge the limitations concerning the understanding, meaning, and applicability of findings in clinical practice. For instance, meta-analyses, in many cases, included RCTs performed on individuals in a variety of pathological settings and the extrapolation of results to the general population is not straightforward.

### Evidence Coming from Recent RCTs

In the last 3 years, evidence generated from RCTs on the effect of plant-based diets has been plentiful. Limiting this list to trials where at least one arm included a recognizable plant-based diet as the main exposure of interest and where BP and/or hypertension were outcomes of interest returned 39 papers (Table 1). Most studies concern the exposure of overweight/obese subjects at (high) risk of cardiometabolic disease, e.g., metabolic syndrome, type 2 diabetes mellitus (T2DM), and non-alcoholic fatty liver disease (NAFLD), to diets based on the Mediterranean or DASH dietary patterns, although vegan and vegetarian diets, among others, were also investigated. Healthy/pathological context scenarios other than the above mentioned include rheumatoid arthritis and chronic kidney disease, etc. The main results of these studies regarding BP values and/or hypertension reinforce the conclusions of the above-mentioned umbrella review and several other meta-analyses. Sub-studies from the PREDIMED and PREDIMED-Plus trials confirm that a high adherence to the Med diet is associated with lower BP values (vide infra). Of note, non-treated participants following a Med diet showed less need for the use of antihypertensive drugs [25]. Indeed, Med diet adherence was associated with decreased need of escalating antihypertensive therapy in patients who were using two drugs at baseline and attenuated the association of antihypertensive drug use with the risk of cardiovascular events [25]. As for the PREDIMED-Plus trial, at baseline, a tendency was found towards a lower Med diet adherence for participants with the highest validated MetS severity score, which (among many other parameters showing unfavorable readings) were characterized by high BP levels [26]. On the other hand, also at baseline, adherence to eight a priori high-quality dietary scores did not show inverse associations with hypertension [27]. Longitudinal analysis showed that the reductions in SBP and DBP were enhanced with higher carbohydrate quality index (categorized in quintiles and based on four criteria: total dietary fiber intake, glycemic index, whole grain/total grain ratio, and solid carbohydrate/total carbohydrate ratio) [28]. Decreases in SBP were significantly associated with increased nut consumption [29], although results from a 2-month crossover RCT showed non-significant intra- and inter-group differences in SBP and DBP in MetS subjects who consumed a Med diet or a non-Med diet plus nuts (50 g/day) [30]. Some studies propose that, alongside a higher adherence to Med diet, other factors, such as physical activity, low sedentary time, or low depression risk, could contribute to enhance the positive effects seen on hypertension (among other CVD risk factors) [31, 32].

Apart from the PREDIMED trial, in the last 3 to 4 years, many RCTs on the effects of Med diets have been published in different physio-pathological contexts. Adherence to Mediterranean-based dietary patterns was associated with favorable effects on BP in apparently healthy individuals [33], overweight/obese at high risk of CVD [34-38], MetS [39], prediabetic [40], T1DM [41], T2DM [42], NAFLD [43, 44], heart or lung transplant recipients [11], obstructive sleep apnea [31], HIV [45], rheumatoid arthritis [46], and cancer patients [47], among others. However, in general, the differences observed were not significant between the intervention groups on the Med diet and other groups. In some cases, neither intra-group nor inter-group significant differences were found, as was the case of individuals without major CVD who consumed the Atlantic diet, for 6 months. This diet has several characteristics in common with the Med diet such as high consumption of vegetables, fruits, whole grains, beans, and olive oil as a key fat source. It is also characterized by high intake of fish and seafood, starch-based products, nuts, milk, and cheese. In professional female handball players who consumed a free diet, a Med diet, or a high antioxidant diet, for 12 weeks, there were no significant differences in BP over time or between groups [48, 49].

Regarding the effects of DASH-based diets on BP, a longitudinal analysis was conducted with one-year data of changes in the DASH diet score and its association with cardiometabolic risk factors in PREDIMED-Plus trial [50]. After adjusting for several potential confounders, higher DASH diet scores were significantly associated with lower SBP (− 0.57 mmHg) and DBP (− 0.15 mmHg). Furthermore, in a RCT with individuals with untreated high normal BP or stage 1 hypertension, Japanese cuisine-based DASH (J-DASH) diets, which are in accordance with the nutritional composition of the DASH diet (but have less total fat and saturated fatty acids), improved home measured BP and stabilized its variability compared to a group who consumed their usual diet [51]. An investigational diet mimicking the average East Asian diet, but retaining common characteristics of the Med and DASH diets, also produced positive effects on BP, in subjects with T2DM [52]. Finally, in CVD risk patients with MetS, a modified DASH diet with additional emphasis on plant-based and Med diets to optimize refeeding also led to a reduction in SBP after 3 months. According to the results of this study, volunteers who had a 5-day fasting prior to following the modified DASH diet showed a sustained reduction both in 24-h ambulatory SBP and mean arterial BP (MABP). Indeed, subjects undergoing fasting reduced their intake of antihypertensive medication in 43% of cases, whereas on DASH alone this happened in 17% of cases.

In the context of RCTs aimed at assessing the effects of vegan and/or vegetarian diets where BP values were evaluated a few studies are worth discussing. In a crossover RCTwith overweight individuals, SBP and DBP values decreased (−9.3 and −7.3 mmHg, respectively) in subjects on a Med diet compared with a vegan diet (−3.4 and − 4.1 mmHg, respectively) [53]. In contrast, 6 months of a vegetarian diet (very-low-protein diet) significantly lowered SBP and DBP more effectively than a Med diet or a Med diet supplemented with essential amino acids and ketoanalogs (Med diet + KA) [54]. In another study, subjects with cardiometabolic risk factors were instructed to consume ad libitum whole-food plant-based diets, consisting of vegetables, grains, legumes, and fruits and avoidance of animal products. Compared to the control group, who consumed an omnivorous diet, there was a tendency for more favorable effects in SBP and DBP [55]. In another study, a low-carbohydrate vegan diet, high in canola oil and plant proteins, was compared to a vegetarian therapeutic diet in type 2 diabetics [56]. There were no changes in BP medications and no treatment differences in BP. However, within treatment, significant reductions in SBP and DBP were seen on the low-carbohydrate vegan (−4.12 and −3.54 mmHg, respectively) and on the vegetarian diet (−5.91 and −4.13 mmHg, respectively). Still on vegetarian diets, a plant-based diet built on the USDA healthy vegetarian meal plan, with modifications to exclude eggs and dairy products, was compared with the same diet except for eggs being permitted (2 eggs/d for 6 weeks while preserving an isocaloric condition) in individuals at risk of T2DM. SBP, DBP, and MABP decreased from baseline in both groups (statistical significance was only found for plant-based diet + eggs), yet changes were not significantly different between groups [57]. On the other hand, a plant-based diet with either 2 eggs/d or the equivalent amount of egg substitute showed no effect in SBP and DBP in MetS subjects [58]. A study carried out in prediabetic subjects who consumed one of three isocaloric-restricted diets: (1) Med diet, (2) a traditional Jiangnan diet high in plants, or (3) a control diet low in plants, for 6 months, showed significant decreases from baseline in SBP and DBP for all diets but differences between groups were non-significant [40]. In a another RCT, rheumatoid arthritis patients completed a 7-day fast followed by an 11-week plant-based diet or a 12-week standard Deutsche Gesellschaft für Ernährung (DGE) diet [59]. Neither dietary protocol lowered SBP, although there was a tendency to decrease DBP in the fasting + plant-based diet group. In another study, all 3 healthy eating patterns: (1) plant-based diet, (2) AHA diet, or (3) Med diet, were associated with similar statistically significant improvements in SBP and DBP, in children with BMI > 95% age/sex predicted [60].

In T2DM [61] and in normotensive women [62], increasing the amount of vegetable, fruit and whole grain dietary intake produced favorable effects in BP. On the other hand, BP did not improve in children, adolescents, and adults included in an intervention group instructed to eat more healthy food items, such as fruits and vegetables, legumes, fish, nuts and seeds, and olive oil, compared to the control group [63]. Furthermore, 12 weeks of consumption of a traditional Brazilian Diet (DieTBra), consisting of plenty of tropical fruits, rice and beans, raw and cooked vegetables and legumes, moderate consumption of dairy products, small portion of red meat, and predominant use of soy oil, did not produce a significant reduction in BP in severely obese individuals (BMI ≥ 35.0 kg/m^2^) [64].

### Plant-Based Diet Components with Hypotensive Actions

#### Vitamin C

Fruits and vegetables are sources of vitamin C, whose effects on BP have been investigated both in clinical trials and in basic biochemistry experiments. Indeed, there is solid evidence from clinical studies that vitamin C treatment restores endothelial function in patients with coronary artery disease or coronary risk factors [65-67]. Of note, there are at least two meta-analyses [68, 69] that conclude that provision of vitamin C (usually 500 mg/d) significantly reduce SBP and DBP and that this effect is more pronounced in hypertensive patients. Yet, it is unfortunate that the near totality of internists or cardiologists do not prescribe ascorbic acid as supplement.

In terms of mechanisms of action, there is clear biochemical evidence that ascorbic acid stimulates endothelium-derived nitric oxide (EDNO) synthesis [70, 71] and that it does so mainly by increasing the intracellular levels of tetrahydrobiopterin, i.e., the most important cofactor for eNOS activity [72, 70]. Even though ascorbic acid is an important antioxidant [73, 74], it does not affect GSH levels in human aortic endothelial cells (HAEC), and it does not increase the GSH/GSSG ratio. Therefore, based on the literature [72, 71], it is unlikely that ascorbic acid directly improves the intracellular redox environment and, consequently, EDNO synthesis. The most conceivable mechanism of action involves the maintenance or even increase of tetrahydrobiopterin levels by reducing dihydro- or trihydrobiopterin back to tetrahydrobiopterin [75], as also shown by basic experiments in which ascorbic acid was found to stimulate purified eNOS activity directly, despite limiting concentrations of tetrahydrobiopterin and without the contribution of GSH [72]. One caveat is that data obtained from in vitro experiments should be interpreted with caution, as culture media are devoid of vitamin C. Indeed, cultured cells are in scorbutic states [76] and exogenous vitamin C first replenishes their stores and then, eventually, exerts biological effects [77].

A critical issue is that of the optimal dose of vitamin C that augments BP. The only complete pharmacokinetic study carried out with vitamin C is that of Levine et al. [78••], who concluded that vitamin C daily doses above 400 mg have no evident value. Also, a review of over 200 articles on vitamin C and health concluded that a daily intake of 100 mg of vitamin C is associated with lower incidence of heart disease, stroke, and cancer [79]. It should be noted that these amounts (1) are consistent with those reported by Levine et al. to saturate cells [78••]; (2) are consistent with the maximal velocity of the vitamin C transporter [80]; and (3) are achievable through a balanced diet [81]. Indeed, it is worth underscoring that, despite some propaganda on mega-doses, ascorbic acid’s concentrations are tightly regulated in the body [82, 83]. One way to overcome such limitations is to administer vitamin C intravenously [84] in order to reach millimolar plasma concentrations. However, the scant clinical trials of intravenous ascorbic acid for cancer treatment yielded negative results [85] and much more well-performed research on a large number of patients is needed to clarify this issue. However, pertinent to this review, Bruno and colleagues demonstrated that acute vitamin C infusion (3 g i.v. in 5 min) significantly lowers BP in hypertensive patients, but not in normotensive subjects [86]. These data confirm those of Heitzer et al. [87], who reported anti-hypertensive effects vitamin C infusion is smokers (whose endothelial function is notoriously impaired). Hence, there might be room for future high-quality trials in hypertensive patients, even though it might be quite impractical to perform ascorbic acid infusions in an outpatient setting.

In summary, ascorbic acid may play a key role in the plant-based diet beneficial effect on BP. These effects are likely not mediated by redox modulation and involve maintenance of cofactors such as tetrahydrobiopterin [88]. Based on the literature, we advocate the use of vitamin C by clinicians and patients as adjunct therapy of hypertension.

#### Potassium

Potassium is the most abundant intracellular mineral, and its role in the regulation of BP is well established [89], even if there is no agreement among authors [90]. Unfortunately, the contribution of potassium to the healthful effects of a plant-based diets is often overlooked [91] in favor of, e.g., (poly)phenols (vide infra). However, many vegetables such as green leafy vegetables and some fruits such as bananas are rich in potassium [92], which might at least in part explain [93] why vegetarian diets are associated with significant reductions in BP compared with omnivorous diets [12]. Historically, humans have been consuming quantities of potassium that were much higher than the current ones [94]. Life expectancy was much shorter, but current potassium intakes are often much lower than the recommended levels [95].

The first clinical evidence of the effects of potassium on BP is likely that of WL Addison [96], and some researchers have been investigating the mechanisms of actions that underlie potassium’s BP-lowering activities [97, 93]. One proposed mechanism involves the stimulatory effects of potassium on the sodium–potassium ATPase, which hyperpolarizes cells [98], hence decreasing cytosolic calcium concentrations and leading to augmented vasomotion. Also, potassium has been suggested to directly stimulate EDNO synthesis [99] and increase natriuresis [99, 91, 97] by inhibiting renal sodium reabsorption.

Finally, we propose an indirect effect of high potassium intake, i.e., that high consumption via a plant-based diet largely replaces that of sodium, either as a natural component of meats (especially smoked and cured meats), some comfort foods, e.g., pizza and savory snacks, or added as part of salt to enhance flavor and taste [100].

#### (Poly)phenols

As mentioned, nitric oxide synthesis can be modulated by dietary factors [101]. Many constituents of plants and, hence, plant-based diets are non-nutritional compounds that play key roles in plant physiology and interactions with the environment [102, 103]. The most actively studied minor components are (poly)phenols, which have been long investigated for their supposed antioxidant activities. Indeed, there is negative correlation between (poly)phenols intake and blood pressure [104]. Even though research has disproven their direct antioxidant actions, their effects on various enzyme activities appear to be even more interesting in terms of health protection: research in this area should be and, indeed, is further extensively promoted [105].

Among the various biological activities of (poly)phenols, their actions on endothelial function have been researched by various groups. Our own group reported the vasomodulating activities of wild plant extracts rich in (poly)phenols (namely wild artichoke and thyme) in vitro [106] and in vivo [107]. Others studied the vasodilator effects of olive extracts, rich in oleuropein, hydroxytyrosol [108, 109], and hypotensive peptides [110, 111] and used as decoction in folk medicine [112, 113].

As mentioned, direct antioxidant, free radical-scavenging activities of (poly)phenols have been ruled out due to their poor bioavailability and reactivity [114•, 115]. However, in biochemical terms, plasma membrane-associated eNOS is considered to be more constitutively active and extremely sensitive to agonist-induced intracellular Ca^+2^ fluxes. In addition to post-translational regulatory mechanisms dictating eNOS function, redox changes in the endothelium do indeed modify both enzyme activity as well as EDNO production, in turn adversely affecting vasomotion [116]. As discussed for ascorbic acid, eNOS synthetic activity is dependent on maintaining tetrahydrobiopterin in a reduced state. When the tetrahydrobiopterin/biopterin ratio is high eNOS produces NO, however, when that redox ratio declines, the internal electron transport chain of eNOS becomes uncoupled, which actually generates superoxide anion rather than NO [117]. Thus, in a pro-oxidative milieu [115], eNOS may not only become a target of oxidant species, but also may exacerbate oxidative stress and endothelial dysfunction, hence impairing vasomotion and leading to hypertension. In brief, even though (poly)phenols are not direct antioxidants, they contribute to maintain a proper endothelial redox status and contribute to the hypotensive actions of a fruit- and vegetable-rich diet.

In addition to epidemiological data (often related to the Mediterranean diet [118]) linking a plant-based diet with lower blood pressure [119], some human studies provided direct evidence of the (poly)phenol-induced increase in vasomotion. Three examples are worth mentioning. One is tea, whose intake (chronic or acute) increases flow-mediated dilation (FMD) in healthy subjects and hypertensive patients [120, 121]. The effects are highly likely due to catechin and its derivatives, given the juxtaposition of their plasma kinetics with FMD [122]. Another (poly)phenol-rich food that has been extensively studied is cocoa/chocolate [123, 124]; again, the most active component appears to be catechin, acting through mechanisms of actions that are only partly elucidated and likely fit with what we described above. Finally, recent research is addressing beetroot juice as functional food with remarkable hypotensive actions [125, 126]. In reason of its vasomodulating actions, beetroot juice is being used as a sport supplement [127] and attributed with ergogenic effects that need to be confirmed by human trials [127, 128].

In summary, (poly)phenols are a likely important contributor to the hypotensive actions of plant-based dietary regimens. Even though the extent and true nature of their biochemical actions has not been fully elucidated, it is very likely that a combination of indirect antioxidant activities (thereby maintaining eNOS function and avoiding its uncoupling), anti-inflammatory actions, direct eNOS hydroxylation, antagonism of L-type Ca^2+^ channels [129], etc. contributes to the augmented vasomotion associated with clinical trials and demonstrated by some human experiments.

#### Omega 3 Fatty Acids

Fatty acids of the omega 3 (or n-3) series, i.e., alfa-linolenic (18:3; ALA), eicosapentaenoic (20:5, EPA), and docosahexaenoic (22:6, DHA), are essential and must be derived from the diet [130]. Their cardiovascular effects have been the subject of much investigation [131] that — from a clinical point of view — led to mostly inconclusive results [132-135].

Among the potentially healthful actions of omega 3 fatty acids, modulation of endothelial function and, hence, of blood pressure has been proposed. The rationale behind the purported activities of omega 3 fatty acids on the endothelium and vasomotion is manifold. For example, omega 3 fatty acids are anti-inflammatory molecules [136] and inflammation greatly participates in the development of, e.g., arterial stiffness [117], impaired vasomotion [137], and hypertension [138]. Moreover, that are — paradoxically — anti- rather than pro-oxidant [139, 140], via molecular mechanisms that have been largely elucidated and include, e.g., inhibition of NADPH oxidase 4 (Nox 4), V sPLA_2_, and cyclooxygenases, as well as reduced superoxide production [140], thereby maintaining an adequate “peroxide tone” [141].

Human studies of the effects of omega 3 fatty acids on vascular tone and blood pressure yielded mixed results. For example, a recent publication by Bischoff-Ferrari et al. [142] reported that administration of 1 g/d of algal oil (330 mg of EPA and 660 mg DHA) had no significant effects in improving systolic and diastolic blood pressures of older adults. Yet, the uncertainty surrounding the hypotensive effects of omega 3 might depend on several factors such as the dose and the choice of patients. Accordingly, a recent dose–response metanalysis by Zhang et al. [143] reported a nonlinear dose–response relationship for systolic and diastolic blood pressure. The computed J-shaped curves implicate that dosages of EPA + DHA between 2 and 3 g/day (combined) were associated with the strongest effects. A slightly older metanalysis published by Guo and coworkers [144] concluded that provision of EPA and DHA leads to a significant reduction in systolic (EPA) and diastolic (DHA) blood pressure in subjects with dyslipidemia or with high C-reactive protein (CRP) concentrations, i.e., at high risk of cardiovascular disease. Whether such effects are due to a direct action of EPA and DHA on the endothelium or to the reduction of CRP concentrations, i.e., anti-inflammatory effects (or a combination of both mechanisms), remains to be elucidated. In this respect, Mori et al. [145] showed that omega 3 fatty acids, in particular DHA rather than EPA, improved vasodilator responses to endogenous and exogenous NO donors and attenuated vasoconstrictor response to norepinephrine in the forearm microcirculation of humans. The mechanisms appeared to be predominantly endothelium independent, based on enhanced vasodilatory responses after the co-infusion of acetylcholine with L-NMMA and the infusion of nitroprusside, both of which are endothelium independent.

As regards ALA (the vegetal omega 3 fatty acid), there are — to the best of our knowledge — no large RCTs exploring its effects on blood pressure, even when given at high doses [146]. However, the use of flaxseed products results in significant reductions of diastolic and systolic blood pressures [147], which might be at least in part due to its high content of ALA. The same applies to a nutraceutical providing Perilla Frutescens powder [148] and to high walnut use by elderly subjects [149]. Mechanistically, it is worth reminding that ALA is converted into oxylipins [150] with biological activities in the vessel wall. In short, epidemiological evidence is strongly suggestive of the cardioprotective actions of ALA [151], which might include modulation of blood pressure [152]. Clinical trials are scant and not concordant, calling for further research on this medium-chain fatty acid.

To summarize, a plant-based diet provides polyunsaturated fatty acids that, when consumed in high proportions, likely exert cardioprotective effects. Whether and to what extent such effects are modulated by augmented vasomotion and BP control is still unclear and requires more research.

#### Omega 6 Fatty Acids

With some exceptions, e.g., foods elaborated with coconut and palm oils, plant-based diets provide a proportionally high amount of omega 6 fatty acids, largely found in seed oils such as safflower, sunflower, and corn oils and in seeds and nuts, e.g., walnuts, almonds, hemp seeds, or cashews [153].

As opposed to their omega 3 counterparts, omega 6 fatty acids, e.g., linoleic acid (LA), cannot be studied as drugs or supplements because their levels of consumption are much higher than those of omega 3. Therefore, most of the data we have on omega 6 and human health come from observational studies. Further, LA is the precursor of arachidonic acid and, consequently, of pro-inflammatory eicosanoids. Hence, the hypothesis was formulated that we should reduce omega 6 consumption to avoid increasing systemic, low-grade inflammation, i.e., one of the major etiological agents in degenerative diseases (vide infra) [154].

This hypothesis has been disproven [155], and some clinical studies of low-to-modest quality did evaluate the effects of PUFA-rich diets on BP. Due to aforementioned difficulties inherent to omega 6 research, such studies should be interpreted as a “replacement” of one class of fatty acids with another one.

Some observational studies did not find any significant effect of higher intakes of omega 6 fatty acids on systolic and diastolic pressures [156-159]. One example is the International Study of Macro-Micronutrients and Blood Pressure, which enrolled 4680 subjects and analyzed the proportion of dietary LA. The authors reported a non-significant, inverse association between LA use and BP (systolic and diastolic) [160]. Yet, a sub-analysis of the control group, i.e., people who were free from cardiovascular diseases or diabetes, did not follow a special diet, and did not take food supplements or medicines, found that BP was modestly yet significantly reduced (− 1.4/ − 0.9 mmHg for each 3.8% more calories from LA) [160]. Other studies [161], nonetheless, did not confirm those data and a metanalysis reported no important effects of omega 6 fatty acids on either systolic or diastolic BP [162].

To summarize, omega 6 fatty acids, namely LA, do not appear to have relevant and clinically-important effects on BP. Therefore, the cardioprotective activities of appropriate omega 6 intakes [163, 164] are likely due to their cholesterol-lowering actions rather than to their effects on BP.

## Conclusions

The data discussed in this systematic review allow us to conclude that plant-based diets are associated with lower BP and overall better health outcomes (namely, on the cardiovascular system) when compared with animal-based diets. The mechanisms of action are being actively investigated and involve many micronutrients plentiful in plants and the dishes prepared with them. We also stress the need to look at human health as closely intertwined with planetary health, as it is necessary to consider the overall “environmental pressure” of food production. In general, animal-based diets have a greater impact than plant-based ones; for this reason, the latter should be promoted in a “one health” framework [165].

## Supplementary Information

Below is the link to the electronic supplementary material.

Supplementary file1 (DOCX 14 KB)

## Data Availability

All data presented are contained within this manuscript.
