# Supplementary material for: Plant-Based Diets Reduce Blood Pressure: A Systematic Review of Recent Evidence
Source: Curr Hypertens Rep. 2023 May 13;25(7):127–50. doi: 10.1007/s11906-023-01243-7 (PMC10224875; doi:10.1007/s11906-023-01243-7)
Supplement: Supplementary file 1 — Supplementary file1 (DOCX 14 KB) [file 11906_2023_1243_MOESM1_ESM.docx]

**Supplementary Table 1.** Literature search strategy used for each database

| **Database** | **Syntax** |
| --- | --- |
| PUBMED | (((("plant-based diet"[Text Word] AND ("hypertension"[Text Word] OR "blood pressure"[Text Word])) OR ("plant-based diet"[Text Word] AND ("hypertension"[Text Word] OR "blood pressure"[Text Word])) OR ("mediterranean diet"[Text Word] AND ("hypertension"[Text Word] OR "blood pressure"[Text Word]))) NOT ("meta analys*"[Text Word] OR "metanalys*"[Text Word] OR "review*"[Text Word])) AND ("english"[Language] AND 2020/01/01:2023/12/31[Date-Publication])) AND ((randomizedcontrolledtrial[Filter])) |
| WEB OF SCIENCE | TS=(("plant based diet") OR ("plant-based diet") OR ("Mediterranean diet") AND (("blood pressure") OR (hypertension))) and Review Article (Exclude – Document Types) and 2023 or 2022 or 2021 or 2020 (Publication Years) and Article (Document Types) and English (Languages) and Randomized Controlled Trial (Search within all fields) and Book Chapters (Exclude – Document Types) |
| SCOPUS | ( TITLE-ABS-KEY ( "plant based diet" AND hypertension ) OR TITLE-ABS-KEY ( "plant based diet" AND "blood pressure" ) OR TITLE-ABS-KEY ( "plant-based diet" AND hypertension ) OR TITLE-ABS-KEY ( "plant-based diet" AND "blood pressure" ) OR TITLE-ABS-KEY ( "Mediterranean diet" AND hypertension ) OR TITLE-ABS-KEY ( "Mediterranean diet" AND "blood pressure" ) ) AND PUBYEAR > 2019 AND PUBYEAR < 2024 AND ( LIMIT-TO ( DOCTYPE,"ar" ) OR EXCLUDE ( DOCTYPE,"ch" ) OR EXCLUDE ( DOCTYPE,"re" ) OR EXCLUDE ( DOCTYPE,"ed" ) OR EXCLUDE ( DOCTYPE,"cr" ) ) AND ( LIMIT-TO ( LANGUAGE,"English" ) ) AND ( LIMIT-TO ( EXACTKEYWORD,"Randomized Controlled Trial" ) ) |
